# At-home bleaching *versus* whitening toothpastes for treatment of tooth discoloration: a cost-effectiveness analysis

**DOI:** 10.1590/1678-7757-2023-0336

**Published:** 2024-02-05

**Authors:** Mariana Evangelista SANTOS, Rênnis Oliveira da SILVA, Yuri Wanderley CAVALCANTI, Sônia Saeger MEIRELES

**Affiliations:** 1 Universidade Federal da Paraíba Programa de Pós-graduação em Odontologia João Pessoa PB Brasil Universidade Federal da Paraíba, Programa de Pós-graduação em Odontologia, João Pessoa, PB, Brasil.; 2 Universidade Federal da Paraíba Departamento de Clínica e Odontologia Social João Pessoa PB Brasil Universidade Federal da Paraíba, Departamento de Clínica e Odontologia Social, João Pessoa, PB, Brasil.; 3 Universidade Federal da Paraíba Departamento de Odontologia Restauradora João Pessoa PB Brasil Universidade Federal da Paraíba, Departamento de Odontologia Restauradora, João Pessoa, PB, Brasil.

**Keywords:** Cost-benefit analysis, Dentifrices, Toothpastes, Carbamide peroxide, Tooth bleaching

## Abstract

**Objectives:**

This study aimed to analyze the cost-effectiveness of whitening toothpastes and at-home bleaching for the treatment of tooth discoloration.

**Methodology:**

A cost-effectiveness economic analysis was conducted, and eight randomized clinical trials were selected based on the whitening agent product used: blue covarine dentifrices (BCD), hydrogen peroxide dentifrices (HPD), dentifrices without bleaching agents (CD, negative control), and 10% carbamide peroxide (CP10, positive control) for at-home bleaching. The consumer/patient perspective was adopted, macro-costing techniques were used and a decision tree model was performed considering the costs in the American and Brazilian markets. The color change evaluation (ΔE*_ab_) was used to calculate the effectiveness of tooth bleaching. A probabilistic analysis was performed using a Monte Carlo simulation and incremental cost-effectiveness ratios were obtained.

**Results:**

CP10 resulted in the highest cost-effectiveness compared to the use of dentifrices in both markets. In Brazil, HPD was more cost-effective than BCD and CD. In the US, the increased costs of HPD and BCD did not generate any whitening benefit compared to CD.

**Conclusions:**

CP10 was more cost-effective than BCD and HPD for tooth bleaching from the perspectives of the Brazilian and American markets. Decision-making should consider the use of CP10 for treating tooth discoloration.

## Introduction

Analytical studies on cost-effectiveness are being performed to establish priorities in healthcare, especially involving the allocation of resources, thus allowing the comparison among different treatment alternatives.^1^ These analyses directly influence decision-making, along with efficacy, effectiveness, adverse effects, and longevity of the treatment protocol.^2^

Tooth bleaching is one of the most common procedures requested by patients who are dissatisfied with their tooth color. However, the high cost of this treatment has stimulated the marketing of over-the-counter (OTC) products for at-home bleaching. OTC products appear to be a low-cost alternative for treating tooth discoloration, requiring no professional supervision and promise tooth whitening with continuous use.^3 - 5^ The literature reports that tooth bleaching is a procedure that influences the patient’s aesthetic self-perception, promoting a positive impact on psychosocial relationships and satisfaction with the appearance of their smile.^6 - 8^ Bleaching treatments can improve the perception of aesthetic areas such as smiling, laughing, and showing teeth without embarrassment in young people.^8^ Another study showed that bleaching procedures were able to improve aesthetic self-perception in adults, especially in the areas of psychological discomfort and social disability.^6^In this context, this treatment is no longer a purely aesthetic procedure, but it is capable to improve the quality of life and personal relationships of young or adult patients. OTC bleaching products are alternatives that arouse the interest of these individuals in whitening their teeth, due to the low cost of these products, ease of purchase, and wide availability on the market.^3 , 5^

Tooth bleaching can be performed in the dental office, where the dentist applies high concentrations of hydrogen peroxide (HP) (25–40%) or carbamide peroxide (CP) (30–37%) to the enamel surface^4 , 9 , 10^ or at-home, where the patient wears a custom tray filled with low concentrations of CP (10–22%) or HP (4–8%) a few hours a day, for a minimum of two weeks^7 , 9^The mechanism of action of bleaching agents is based on the HP oxireduction process, which degrades HP into hydroxyl, perhydroxyl free radicals, and superoxide anions.^11^ Due to its low molecular weight, HP diffuses into dentin, promoting the breakdown of carbonic double bonds of unsaturated organic molecules into saturated components, subsequently modifying their optical properties.^11^

A wide variety of OTC bleaching products, such as dentifrices, mouth rinses, whitening strips, dental floss, gums, varnishes, and whitening toothbrushes are widely available to consumers in supermarkets, pharmacies, and e-commerce.^5 , 12 , 13^ Whitening dentifrices represent more than 50% of OTC products and contain different bleaching components ranging from abrasive agents (hydrated silica, silicon dioxide, calcium carbonate, and activated carbon),^5 , 14^ enzymatic activities (bromelain and papain),^15^and particles with optical effects (blue covarine)^3 , 5^ to low concentrations of CP or HP.^14 , 16^ Insoluble abrasives remove extrinsic stains from the tooth surface.^5^ The blue covarine pigment acts with optical effect due to its ability to modify the way the light is reflected on the tooth by depositing the blue pigment on the tooth surface.^3 , 5 , 16^ However, the bleaching ability of blue covarine is controversial. While previous studies have reported that the presence of this pigment promotes a clinically perceptible color change,^14 , 17^ others have demonstrated that the use of these dentifrices did not improve tooth color.^3 , 13^

The high cost of tooth bleaching procedures makes it a treatment modality that is difficult to access and correlates the demand for this service to the population’s purchasing power, which can vary according to the region or country in which it is performed. According to data provided by the World Bank in 2020, the United States (US) had a gross domestic product (GDP) per capita of $63.416, whereas Brazil had a GPD per capita of $6.797.^18^Willingness to pay (a parameter used in cost-effectiveness analysis) is the maximum price that a consumer will pay for a service and varies according to the country and criteria adopted. For example, in the Brazilian scenario, there is no definition of the value that should be attributed to willingness to pay.^1 , 19 , 20^

Thus, it is necessary to perform a health economic assessment to compare the different alternatives to OTC and low-cost bleaching products. This analysis was carried out in a systematic and objective way, considering the financial costs of bleaching products and their consequences, and estimating a direct relationship in monetary terms and health outcomes.^19^ Although whitening toothpastes are widely available for self-consumption, there are few randomized clinical trials that assess the bleaching effectiveness of these products.^3 , 21^ Additionally, no study has evaluated the cost-effectiveness of these products for bleaching treatments.

Thus, this study aimed to perform a cost-effectiveness analysis, from the consumer/patient perspective, of two tooth bleaching technologies: supervised at-home bleaching and whitening dentifrices. The null hypothesis tested was that there was no difference between the cost-effectiveness of whitening dentifrices and at-home tooth bleaching.

## Methodology

### Study design

A full economic cost-effectiveness analysis was designed to evaluate the competing alternatives for the same outcome. The products used for this analysis were blue covarine-based dentifrices (BCD), 0.75–2.8% hydrogen peroxide dentifrices (HPD), conventional dentifrices without bleaching agents (CD, negative control), and at-home bleaching with 10% carbamide peroxide (CP10, positive control) ( Table 1 ).

**Table 1:** Groups according to the bleaching agents and mechanism of action

Groups	Main agent	Mechanism of action
BCD	Blue covarine	Deposition of blue pigment on the tooth surface modifies the interaction of light with the tooth structure.
HPD	Hydrogen peroxide	Removal of intrinsic stains by breaking dental pigmentation through an oxidation-reduction process.
CD	Conventional toothpaste	There is no bleaching agent.
CP10	10% carbamide peroxide at-home bleaching	Removal of intrinsic stains by breaking dental pigmentation through an oxidation-reduction process.

The economic evaluation of this study followed the methodological guidelines of the Consolidated Health Economic Evaluation Reporting Standards (CHEERS)^21^ which are defined as analytical techniques that compare different alternatives, weighing up the costs and their consequences for health, whether negative or positive. This cost-effectiveness analysis used computer modeling based on randomized clinical trials that related resource consumption and health outcomes. The problem studied was: “What is the cost-effectiveness of dentifrices with bleaching agents on tooth color change?” The question was answered using a population consisting of patients over the age of 18 who used dentifrices; the intervention, tooth brushing with whitening dentifrices; the comparison, 10% CP at-home bleaching and CD without bleaching agents; and the outcome, tooth bleaching. The articles included in this analysis were subjected to peer review.

### Population perspective

The perspective adopted for this analysis was that of the consumer/patient.

### Interventions

The interventions to solve the problem in the decision tree were tooth brushing with whitening dentifrices, dentifrices without whitening agents (negative control), and CP10 at-home bleaching (positive control) performed in adult participants who participated in randomized clinical trials in 2000 and 2021. These studies used the CIEL*a*b* color system as the method to evaluate bleaching effectiveness. Tooth color was determined according to the CIEL*a*b* coordinates, where L* represents the value ranging from 0 (black) to 100 (white), a* represents the value of redness (positive a*) to greenness (negative a*), and b* represents the value of yellowness (positive b*) or blueness (negative b*).^19^ Tooth bleaching typically occurs by increasing the lightness (higher L*) and reducing the yellowness (lower b*) of the tooth structure. The formula ΔE*_ab_= 12.3 was used to calculate the difference between the color coordinates.^22^

### Discount rate and time horizon

An annual fee of 5% was applied for the costs and expenses. A time horizon of 12 months was considered, defined according to the most used evaluation period in the randomized clinical trials included in this study.

### Model structure

**Figure 1:**
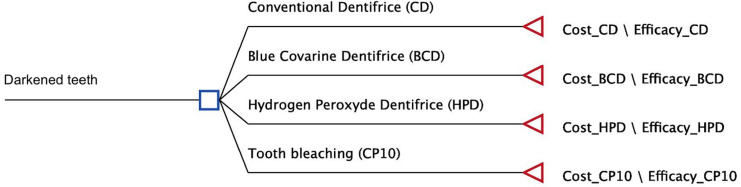
Decision tree

The decision tree model was chosen for this analysis because it represents clinical problems and is directly related to short-term outcomes ( Figure 1 ). Moreover, it is a visual tool used to make decisions on health-related issues. The decision tree comprises three main components: model, probabilities of occurrence of various events, and outcome values. A decision tree model was designed, considering the monetary conversion from the Brazilian market currency (BRL) to dollar (USD), and considering the American market currency (USD) for the Brazilian and American perspectives, respectively. The American market was chosen because it is one of the largest consumer markets for over-the-counter bleaching products in the world and the American dollar is the most widely used currency in international economic transactions. The Brazilian market is a large consumer market and represents the economic reality of the authors of this research.

The structure of this model is made up of nodes, branches, and outcomes. The decision node is represented by a square and indicates the fundamental decision: treatment employed, whitening toothpaste, conventional toothpaste, or at-home bleaching. The terminal node, represented by a triangle, indicates the outcome value.^23^This study used a decision tree to guide the analysis of cost and effectiveness calculations.^23^

### Model input parameters

#### Effectiveness measure

Data were obtained from previously published randomized clinical trials to identify the bleaching effectiveness of the products evaluated in this cost-effectiveness analysis. ΔE*_ab_ values were obtained using a spectrophotometer, colorimeter, or polarized digital images during the following evaluation periods: two or three weeks and 12 months.

#### Costs

The costs of dentifrices and at-home bleaching were measured using the macro-costing technique. For dentifrices, the direct costs of each product were included.^20 , 23^ The values of dentifrices were collected from three websites on the Brazilian market: (https://www.amazon.com.br/, https://www.americanas.com.br/, and https://www.submarino.com.br/), and the American market: (https://www.amazon.com/, https://www.walmart.com/, and https://www.ebay.com/). Due to the ease of access and increased consumption of online products, we decided to collect the toothpastes prices from marketplaces with a wide reach and population use in Brazil and the US. The average price of each product was then calculated. The cost of toothpaste was estimated considering the use of one tube per month over 12 months.

For at-home bleaching performed in Brazil, the Brazilian Hierarchical Classification of Dental Procedures (CBHPO) 2020 was used to obtain the average cost (USD) of the treatment^24^ In the US, we found no references with a hierarchical classification of costs of dental procedures as in Brazil. We therefore decided to use the values available on American websites about supervised at-home bleaching to obtain an estimate of the cost (USD) of the procedure in this market (https://www.dentaly.org/us/teeth-whitening/teeth-whitening-cost/, https://castlevalleydental.com/how-much-does-teeth-whitening-cost-in-the-usa, https://www.yourdentistryguide.com/professional-whitening/). The three websites were selected at random and represent American organizations or dental clinics that operate within the US and charge approximately the same amount for at-home supervised tooth bleaching. The cost charged for at-home tooth bleaching was the same regardless of the type of peroxide and the concentration of the agent. The value used to convert the costs from BRL to USD was 5.42 BRL to 1.00 USD.

## Main assumptions

This analysis aimed to estimate the differences in the costs and effectiveness of whitening products and to calculate the incremental cost-effectiveness ratios (ICERs). To this end, the following assumptions were considered:

1. Tooth brushing (with whitening dentifrices or negative control) was performed 2–3 times/day for twelve months, one tube per month being considered;
2. Professionally supervised at-home tooth bleaching with CP10 was performed for 2 or 3 hours/day for two or three weeks;
3. The American and the Brazilian markets (USD) were considered for the cost-effectiveness analysis.^23^

## Analysis

A probabilistic sensitivity analysis was performed with Monte Carlo simulation using a hypothetical cohort of 1,000 participants to obtain cost-effectiveness acceptability curves to summarize the uncertainty in the cost-effectiveness and facilitate decision-making. The cost-effectiveness of using dentifrices with whitening agents was evaluated and compared with using the at-home bleaching technique with custom trays. Acceptability curves for cost-effectiveness, cost-effectiveness dispersion, net monetary benefit, and decision trees were generated using TreeAge Pro 2021 (TreeAge software, Williamstown, MA).

The Monte Carlo simulation considered the mean and standard deviation values of the cost and effectiveness parameters used in the model and in the generation of the hypothetical cohort. For comparison purposes, the cost and effectiveness of the treatments were ordered from the lowest to the most onerous. ICERs were calculated by dividing the difference in treatment costs and effectiveness.

## Included studies

To select the included studies, a search was performed from January 2021 to March 2022 on Pubmed, using the following keywords: dentifrice; dental polishes; toothpastes; toothpaste; tooth bleaching; teeth whitening; whitening, teeth; tooth whitening; whitening, tooth; teeth bleaching; bleaching, teeth; clinical trial. Using the following search strategy, 128 articles were found: (((Toothpastes) OR (Dentifrices) OR (Dental polishes) OR (Polishes Dental) OR (toothpastes) OR (toothpaste). AND ((randomizedcontrolledtrial[Filter]) AND ( 2000/1/1:2021[pdat]))) AND ((Randomized Controlled Trials as Topic) OR (Clinical Trials Randomized) OR (Trials, Randomized Clinical) OR (Controlled Clinical Trials, Randomized) OR (Randomized Controlled Trial) OR (clinical trial, controlled) AND ((randomizedcontrolledtrial[Filter]) AND (1/1/2000:2021[pdat])))) AND ((Tooth bleaching) OR (Bleaching, Tooth) OR (Teeth Whitening) OR (Whitening) , Teeth) OR (Tooth Whitening) OR (Whitening, Tooth) OR (Teeth Bleaching) OR (Bleaching, Teeth) AND ((randomizedcontrolledtrial[Filter]) AND (2000/1/1:2021[pdat]))), and after evaluating the abstracts, eight randomized clinical trials were included in this study, according to the inclusion criteria. The randomized controlled clinical trials included in this cost-effectiveness analysis study are described in Table 2 .

**Table 2:** Randomized clinical trials included in the cost-effectiveness analysis

Author, year	Intervention	ΔE*_ab_	Sample size	Product (Brand)
Vladislavic, et al.^32^ (2022)	Toothbrushing with hydrogen peroxide dentifrice	3.8	20	Colgate Max Expert White (Colgate-Pamolive, NY, USA)
Kim, et al.^16^ (2020)	Toothbrushing with hydrogen peroxide dentifrice	3.2	15	Toothwhole white (Toothwhole white, Nobldaum, Seoul, Korea)
		4.26	15	Vussen 7 (Vussen 7, Osstem, Seoul, Korea)
		4.26	17	Vussen 28 (Vussen 28, Osstem, Seoul, Korea)
Meireles, et al.^3^ (2021)	Toothbrushing with blue covarine dentifrice	1.4	25	Close Up White Now (Unilever, Ipojuca, Brazil)
	Toothbrushing with conventional dentifrice	1.4	25	Colgate Máxima Proteção Anticáries (Colgate-Palmolive, São Bernardo do Campo, Brazil)
	At-home bleaching with 10% carbamide peroxide	9.9	25	Whiteness Perfect 10% (FGM Dental Products, Joinville, Brazil)
Meireles, et al.^4^ (2021)	At-home bleaching with 10% carbamide peroxide	11.4	20	Polanight 10% (SDI Limited, Bayswater, Victoria, Australia)
Martini, et al.^9^ (2021)	At-home bleaching with 10% carbamide peroxide	8.5	46	Opalescence™ PF 10% (Opalescence, Ultradent Products)
Lópe Darriba, et al.^35^ (2017)	At-home bleaching with 10% carbamide peroxide	5.77	25	Vivastyle Vivadent (Ivoclar Vivadent, Schaan, Liechtenstein)
Meireles, et al.^36^ (2009)	At-home bleaching with 10% carbamide peroxide	4.3	46	Whiteness Perfect 10% (FGM Dental Products, Joinville, Brazil)
Meireles, et al.^25^ (2010)	At-home bleaching with 10% carbamide peroxide	4.0	46	Whiteness Perfect 10% (FGM Dental Products, Joinville, Brazil)

## Results

### Cost-effectiveness ratio

**Figure 2:**
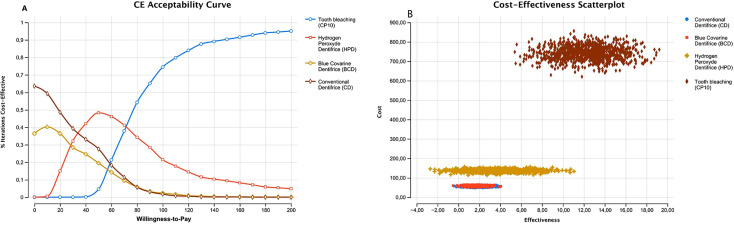
(A) Acceptability curve; (B) Cost-effectiveness scatterplot for bleaching treatments in the Brazilian market

**Figure 3:**
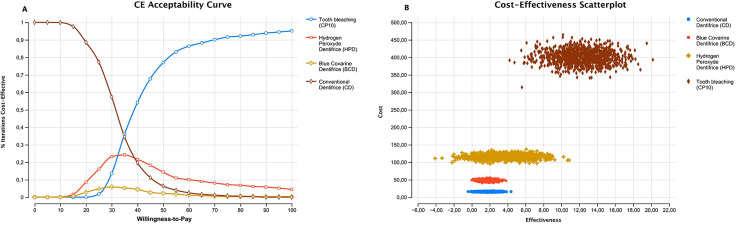
(A) Acceptability curve; (B) Cost-effectiveness scatterplot for bleaching treatments in the American market

The results of the cost-effectiveness analysis are presented in Tables 3 and 4 . Despite the high cost of treatment, CP10 showed higher bleaching effectiveness, resulting in higher net monetary benefit in both markets evaluated ( Tables 3 and 4 , Figures 2A , and 3A ), whereas BCD was dominated in the two markets analysis. In Brazil, the cost-effectiveness of BCD was similar to that of CD, and in the US, the cost-effectiveness of BCD was lower than that of CD ( Tables 3 and 4 , Figures 2B and 3B ). Tooth brushing with BCD showed no whitening benefit compared to CD in both the Brazilian and American markets. Additionally, there was a negative effect of using BCD compared to CD of -5.5 % and -51.2%, for the Brazilian and American markets, respectively ( Tables 3 and 4 ).

**Table 3:** Cost-effectiveness analysis in the Brazilian market

Dominance	Strategy	Cost USD	Incremental Cost	Effectiveness	Incremental Effectiveness	ICER	NMB	%NMB Gain
Not dominated	CD*	10.3	-	1.9	-	-	84.2	0%
Dominated	BCD	10.6	0.3	1.8	-0.08	-3.7	79.6	-5.0%
Dominated	HPD	24.9	14.6	3.7	1.8	7.8	162.9	-93.4%
Not dominated	CP10*	136.3	111.3	12.2	8.5	13.1	476.6	465.5%

*WTP- willingness to pay (USD 50). NMB- net monetary benefit (Effectiveness*WTP – cost). ICER- incremental cost- effectiveness ratio. *Dominant technology. %NMB Gain compared to CD. For the cost conversion, 5.42 BRL was used for 1 USD.

**Table 4:** Cost-effectiveness analysis in the American market

Dominance	Strategy	Cost USD	Incremental Cost	Effectiveness	Incremental Effectiveness	ICER	NMB	%NMB Gain
Not dominated	CD*	15.8	-	1.9	-	-	80.4	0%
Dominated	BCD	48.3	32.4	1.7	-0.1	-185.1	36.1	-51.2%
Dominated	HPD	115.9	100.1	3.8	1.9	52.4	75.7	-5.8%
Not dominated	CP10*	400.1	384.3	12.3	10.4	36.7	219.5	172.8%

WTP- willingness to pay (USD 50). NMB- net monetary benefit (Effectiveness*WTP – cost). ICER- incremental cost- effectiveness ratio. *Dominant technology. %NMB Gain compared to CD.

In the Brazilian market, HPD was more cost-effective than the other dentifrices evaluated, resulting in a 93.4% increase in whitening benefit compared to CD ( Table 3 , Figure 2B ). CP10 provided a whitening effect four times greater than HPD, resulting in a 398.4% increase in whitening benefit ( Table 3 ). Moreover, CP10 resulted in a whitening benefit of 465.5% compared to CD ( Table 3 ).

In the US market, CP10 showed the highest cost-effectiveness, predominating over the other dentifrices ( Table 4 ). BCD and HPD were considered dominated, as the benefit achieved with the use of these products was not compatible with the increase in cost compared to CD ( Figure 3B ). CP10 showed a 172.9% increase of whitening benefit when compared to CD. Considering a time horizon of 12 months, BCD showed a higher cost and lower effectiveness than CD ( Table 4 ).

### Cost-effectiveness, probabilistic sensitivity analysis, and decision tree

The cost-effectiveness analysis and acceptability curve showed that CP10 at-home bleaching presented the best cost-effectiveness ratio. In the Brazilian market, HPD was the most cost-effective dentifrice, whereas CD and BCD showed similar cost-effectiveness ( Figure 2B ). Higher percentages of cost-effective interactions and net monetary benefits were observed when the willingness to pay for tooth bleaching was higher than 15.00 USD ( Figure 2A ). In other words, if 15.00 USD out-of-pocket money is feasible for the individual to achieve tooth bleaching, CP10 is a worth option. Furthermore, there was a higher cost and effectiveness for CP10, followed by HPD for cost-effectiveness dispersion ( Figure 2B ).

In the American market, the cost-effectiveness acceptability curve showed that above a willingness to pay value of approximately 35.00 USD, CP10 significantly increased the percentage of cost-effective interactions and the net monetary benefit over the other products evaluated ( Figures 3A and 3B ).

## Discussion

This study was the first to compare the cost-effectiveness of whitening dentifrices and the CP10 at-home bleaching for tooth bleaching procedures performed in the Brazilian and American markets. A cost-effectiveness analysis aims to evaluate the maximum health benefits from the available resources of alternative interventions and to define their potential effectiveness.^1 , 19^ This analysis is a tool that can help in making health decisions, as well as focusing on other factors, such as patient expectations and ethical, cultural, and political concerns.^2^ Additionally, it allows a clear understanding of the compensation between costs, harms, and benefits among treatments using a single metric.^1^ The null hypothesis tested in this study was rejected, since the economic analysis showed that CP10 resulted in a higher cost-effectiveness ratio in both the Brazilian and American markets. Thus, to obtain a clinically significant whitening effect, patients will need to make a major financial investment, as CP10 is the most expensive of the treatments. Considering a time horizon of 12 months,^9 , 25^ tooth color change (ΔE*_ab_ = 12.3) resulting from CP10 at-home bleaching was clinically perceptible (ΔE*_ab_> 1.2) and acceptable (ΔE*_ab_> 2.7).^24^ Moreover, CP10 was chosen as the control group because its effectiveness and longevity are well reported in the literature.^10 , 22 , 25 , 26^

According to CBHPO 2020, the cost of at-home bleaching for the upper and lower dental arches in Brazil was 136.4 USD (739.5 BRL), which corresponds to 61% of the Brazilian minimum wage of 223.6 USD (1.212 BRL) in 2022 and 26.5% of the average Brazilian income of 514.2 (2.787 BRL).^24 , 26 - 28^ In the US, this procedure is financially more lucrative, as it costs around 400.00 USD; the minimum wage was 7.25 USD per hour or 1.160 USD per month, which can be even higher depending on the US state we are referring.^29^ The U.S. Department of Labor reported in 2022 that the weekly income of salaried workers was 1.085 USD, so the cost charged for at-home supervised bleaching in the US corresponded to 36.9% of the average American income.^30^ Despite the high cost, the literature reports that the longevity of CP10 at-home bleaching is up to 30 months.^26^ Thus, investing in this treatment becomes more financially attractive for the patient in terms of willingness to pay, since the cost would be diluted over the longevity of the treatment. Furthermore, the choice of supervised at-home bleaching with CP10 as a positive control was due to the fact that it is an at-home treatment that depends directly on patient compliance, just like the whitening dentifrices included in this study. Generally, clinical trials that evaluate the effectiveness of whitening toothpastes compare them with at-home bleaching using CP10.^3 , 31^ However, we did not include in-office bleaching in this cost-effectiveness analysis, as it is a procedure that depends directly on professional skill, is carried out in the dental office, and costs significantly more than at-home bleaching^4^.

In the two markets evaluated, the treatment with BCD was dominated because the benefits provided were not compatible with the increase in cost in relation to the control dentifrice. The literature is controversial regarding the whitening effect promoted by brushing with BCD. Few studies have reported that the use of these products promotes an improvement in tooth color,^17^ whereas other studies have shown that the color change promoted by the presence of blue pigment is not clinically perceptible.^3 , 13^This study showed that BCD and CD effectiveness were clinically noticeable, but not clinically acceptable.^22^ This analysis showed that a dentifrice without bleaching active can generate a change in tooth color above the clinical perceptibility limit, which cannot be interpreted as tooth bleaching or considered a treatment option.

In the Brazilian market, the use of BCD and CD demonstrated similar cost-effectiveness. In the US market, the cost-effectiveness ratio of BCD was lower than that of CD. Thus, the whitening effect of BCD was inferior to that of CP10 and similar to that of CD and may be related to the mechanism of action of the blue pigment present in BCD. The blue pigment does not hold the ability to remove extrinsic and intrinsic stains, but acts by depositing a thin layer of pigment on the tooth surface, which modifies the light reflection, causing an optical effect of apparently lighter teeth and not a real change in tooth color.^13 , 17^ In the American market, the cost of BCD is three times higher than that of CD, whereas in the Brazilian market, it is similar to the cost of CD. Therefore, monetary investment in BCD is not of interest, as the whitening effect generated using this product is clinically irrelevant.

Although the use of HPD has demonstrated effectiveness above the clinical limits of perceptibility and acceptability, in the US, this technology was considered a dominated procedure, and this can be attributed to the difference in treatment costs in the American market compared to the Brazilian market. The HPD showed a ΔE*_ab_ only 1.8 units higher than that of CD, at a cost approximately 7.5 times higher (additional cost of 100.00 USD). Furthermore, when compared to CP10, the difference in cost between the products was around 285.00 USD and 8.4 units of ΔE*_ab_. In the Brazilian market, HPD was the most cost-effective of the toothpastes evaluated in this study. However, the cost and effectiveness of HPD were significantly lower than that of CP10. Some studies have reported that brushing with HP-based toothpastes can improve tooth color with continuous use^16 , 32^ and this has been attributed to the ability of peroxides to diffuse into the tooth structure, promoting the oxidation of organic molecules responsible for pigmentation, which is similar to the mechanism of action of CP10.^14 , 16^

The difference in the bleaching effectiveness between HPD and CP10 at-home bleaching can be attributed to the higher concentration of the gel and the longer exposure time (2–3 h) of the oxidizing agent during at-home bleaching on the enamel surface. HPD holds a low concentration of HP (0.7–2.8%) and a short contact time with the tooth surface (2–3 min).^3 , 32^Although studies have reported the existence of a whitening effect after using HPD, a randomized clinical trial observed a reduction in effectiveness after continuous use of HPD for 30 days (ΔE*_ab_= 3.7) and 60 days (ΔE*_ab_= 2.9). Additionally, bleaching longevity was lower when the use of the product was discontinued after 30 days of treatment completion.^32^Thus, this product is not financially attractive due to its high cost, low effectiveness, and low longevity, compared to CP10 which holds higher effectiveness and longevity up to 30 months.^26^

In this cost-effectiveness analysis, the cost of toothbrushes was not considered, as the patient used this item daily, regardless of the use of whitening toothpastes. Additionally, the assumption of one tube of toothpaste used per month for one year was used for standardization purposes, since the studies included in this analysis held brushing protocols of 2x or 3x/day. Additionally, only one study included in this financial analysis reported the amount of dentifrice used in tooth brushing, which is 22.9 g for conventional dentifrices and 18.5 g for BCD in a 2x/day brushing protocol for 2 weeks.^3^ Thus, twice this amount would be equivalent to the amount used per month for brushing teeth, which is close to a 50 g tube. For the 3x/day protocol, the equivalent amount of toothpaste used was 70 g. Although tooth sensitivity is an adverse effect frequently associated with tooth bleaching, its prevalence is significant when tooth bleaching is performed with high bleaching agent concentrations.^33 , 34^The treatments analyzed in this study were carried out with low peroxide concentrations. Therefore, we did not include the monetary costs related to preventing tooth sensitivity.

The whitening effectiveness of toothpastes containing bleaching agents has been frequently evaluated in *in vitro* studies, and this analysis prioritizes the selection of randomized clinical trials.^3 , 9 , 17^Moreover, this study excluded other dentifrices with bleaching agents due to the lack of randomized clinical trials evaluating their effectiveness with a quantitative system for color change. The randomized clinical trials included in this analysis considered the CIELAB color system to assess the color change (ΔE*_ab_) resulting from CP10 at-home bleaching or continuous use of BCD or HP whitening dentifrices.^3 , 10 , 16 , 32 , 35 , 36^ This factor can be considered a limitation of this study, as the *Commission Internationale de l’Eclairage* (CIE) currently recommends the use of CIEDE2000 (ΔE_00_) owing to the incorporation of hue and chroma corrections in the ΔE*_ab_ formula of the CIELAB system, whereas aiming to achieve the highest agreement between the shade resulting from the smallest color difference and what is visually observed.^22^However, due to the lack of randomized clinical trials evaluating the bleaching effectiveness of the products employed in this study with the CIEDE2000, we chose to include studies that used the CIELAB system to verify color changes using spectrophotometers, colorimeters, or digital images.^3 , 9 , 10 , 13 , 24 , 32^

The color change associated with whitening dentifrices is usually limited to the ability to remove extrinsic stains, which may be related to the presence of abrasive particles also present in conventional dentifrices.^3^Although a slight improvement in tooth color has been reported, the continuous use of whitening toothpastes in most cases is associated with low bleaching effectiveness.^14^ Even when a color change is observed above the limits of acceptability and perceptibility in the CIELAB system,^22^ the total color change is clinically insignificant when considering the cost invested or when compared with the whitening effect obtained by supervised in-office or at-home bleaching.^3 , 15 , 32^ This study demonstrated that investing in BCD or HPD did not present a good cost-effectiveness ratio for tooth bleaching, as CP10 proved to be more efficient for treating darkened teeth. Furthermore, a larger number of randomized clinical trials are needed to evaluate the effectiveness of toothpastes with different whitening mechanisms, as well as their long-term use.

## Conclusions

Within the limitations of this study, it was concluded that CP10 at-home bleaching showed the highest level of cost-effectiveness when compared to the use of BCD or HPD for tooth bleaching over a 12-month period in the Brazilian and American markets. Financial investments in BCD or HPD did not prove viable for tooth whitening procedures, since the cost of the products was high, considering the low bleaching effect achieved.
